# Resting-state magnetoencephalographic oscillatory connectivity to identify patients with chronic migraine using machine learning

**DOI:** 10.1186/s10194-022-01500-1

**Published:** 2022-10-03

**Authors:** Fu-Jung Hsiao, Wei-Ta Chen, Li-Ling Hope Pan, Hung-Yu Liu, Yen-Feng Wang, Shih-Pin Chen, Kuan-Lin Lai, Gianluca Coppola, Shuu-Jiun Wang

**Affiliations:** 1 Brain Research Center, National Yang Ming Chiao Tung University, Taipei, Taiwan; 2 School of Medicine, National Yang Ming Chiao Tung University, Taipei, Taiwan; 3 Department of Neurology, Neurological Institute, Taipei Veterans General Hospital, 201, Shih Pai Rd Sec 2, Taipei, Taiwan 11217; 4 Department of Neurology, Keelung Hospital, Ministry of Health and Welfare, Keelung, Taiwan; 5 Department of Medico-Surgical Sciences and Biotechnologies, Sapienza University of Rome Polo Pontino, Latina, Italy

**Keywords:** Chronic migraine, Resting-state oscillatory connectivity, Pain disorders, Magnetoencephalography, Machine learning

## Abstract

To identify and validate the neural signatures of resting-state oscillatory connectivity for chronic migraine (CM), we used machine learning techniques to classify patients with CM from healthy controls (HC) and patients with other pain disorders. The cross-sectional study obtained resting-state magnetoencephalographic data from 240 participants (70 HC, 100 CM, 35 episodic migraine [EM], and 35 fibromyalgia [FM]). Source-based oscillatory connectivity of relevant cortical regions was calculated to determine intrinsic connectivity at 1–40 Hz. A classification model that employed a support vector machine was developed using the magnetoencephalographic data to assess the reliability and generalizability of CM identification. In the findings, the discriminative features that differentiate CM from HC were principally observed from the functional interactions between salience, sensorimotor, and part of the default mode networks. The classification model with these features exhibited excellent performance in distinguishing patients with CM from HC (accuracy ≥ 86.8%, area under the curve (AUC) ≥ 0.9) and from those with EM (accuracy: 94.5%, AUC: 0.96). The model also achieved high performance (accuracy: 89.1%, AUC: 0.91) in classifying CM from other pain disorders (FM in this study). These resting-state magnetoencephalographic electrophysiological features yield oscillatory connectivity to identify patients with CM from those with a different type of migraine and pain disorder, with adequate reliability and generalizability.

**Supplementary Information:**

The online version contains supplementary material available at 10.1186/s10194-022-01500-1.

## Introduction

Migraine, a highly prevalent neurological disorder, is a disabling disease that affects more than one billion people worldwide, with a global age-standardised prevalence of 14.4%, according to the World Health Organization’s Global Burden of Disease study [1]. Patients with migraine experience substantial functional disability, especially when the migraine evolves from episodic migraine (EM) to chronic migraine (CM; ≥ 15 headache days per month for > 3 months) [2], which also imposes a considerable economic burden [3]. Migraine is a complex brain network disorder with a strong genetic basis in which interactions of multiple neuronal systems cause the wide variety of symptoms, including pain, that characterise a migraine attack. Neuroimaging studies have revealed widespread structural and functional abnormalities in the brain areas involved in multisensory, affective, and cognitive processing [4–9]. Whether these neuroimaging results represent a potential migraine signature that can be used to discriminate patients with migraine from those without migraine or with other pain disorders remains debatable.

Although many neuroimaging studies have focused on identifying brain signatures for migraine, neurologists still rely on traditional diagnostic tools to diagnose patients with CM. This may be because neuroimaging studies have typically reported the differences between groups, whereas doctors in clinical settings must make clinical decisions at the individual level [10]. To utilise the strengths of neuroimaging for individualised diagnosis of migraine, the supervised machine learning (ML) approach may be a promising method, in which algorithms and techniques are developed to automatically detect patterns in data, which are then used to predict or classify future data. This approach may thus allow a high level of individual characterisation suitable for routine clinical use.

In this study, we used magnetoencephalography (MEG) to directly record neural activity in a wide frequency range and analyse resting-state oscillatory connectivity within cortical networks that are promising for the investigation of ongoing processing in chronic pain [11] and consequently can help diagnose migraine. Compared with conventional scalp electroencephalography, MEG is superior in the localisation and measurement of cortical activities [12]. Notably, although many migraine researches using functional magnetic resonance imaging (fMRI) pointed to the brainstem and hypothalamus as involved in generation and maintenance of migraine attacks [13, 14], we focused on the multisensory higher cortical networks for their engagement of subsequent pain processing. MEG recording, which has excellent temporal resolution and is especially sensitive to neural signals tangential to the scalp, well detects the dynamics of multisensory higher cortical interactions. In contrast, due to the limited temporal resolution, fMRI cannot provide the cortical dynamics in the > 0.1 Hz frequency range. To identify the electrophysiological features for CM diagnosis, we used a classification model to differentiate patients with CM from healthy controls (HC), validated this model with a new testing data set (CM vs. HC), and assessed its performance on another testing data set (CM vs. EM and CM vs. fibromyalgia [FM]) to evaluate generalizability.

## Materials and methods

### Participants

All participants were 20–60 years old, right-handed, had no history of systemic or major neurological diseases, had normal results on physical and neurological examinations, and were enrolled from the headache clinic of Taipei Veterans General Hospital. Patients with EM and CM were diagnosed according to the *International Classification of Headache Disorders, Third Edition* [2], and they were naïve to preventive treatment of migraine. CM was characterized with headache occurring on 15 or more days/month for more than 3 months, which, on at least 8 days/month, has the features of migraine headache. We excluded patients with headache medication overuse and those taking migraine prophylactic drugs, hormones, or other medications on a regular (daily) basis. FM was diagnosed according to the modified 2010 American College of Rheumatology criteria [15]. For the FM group, patients who had a CM, tension-type headache or autoimmune rheumatic disease or who took hormones or other medications on a daily basis were excluded. None of the HC participants had personal or family histories of pain disorders or had experienced any significant pain condition over the previous year. The hospital’s Institutional Review Board approved the study protocol (VGHTPE: IRB 2015–10-001BC), and all participants provided written informed consent before study commencement.

### Study design

All participants completed semistructured questionnaires on demographic information and psychometric scales, including the Hospital Anxiety and Depression Scale (HADS) [16]. For patients with migraine, the headache profile was recorded, including the headache days per month, duration (months) of headache attack, and average headache intensity over the preceding year. Moreover, the Migraine Disability Assessment (MIDAS) questionnaire was administered to evaluate migraine-related disability [17]. All patients maintained a headache diary after recruitment, in which they recorded the following data: date/time of headache attacks, pain intensity, associated symptoms, medication used (if any), and menstrual periods. For patients with FM, the FM profile was collected, including the distribution (widespread pain index) accompanying somatic or psychiatric symptoms (symptom severity scale) [15], and the days of painkiller use per month. In addition, the Fibromyalgia Impact Questionnaire-revised was administered to assess functional disability associated with FM.

Each participant underwent MEG recording. For patients with migraine, the recording was conducted during the interictal period, arbitrarily defined as the absence of an acute migraine attack in the 2 days before and after the MEG recording. The presence of a background or interval headache during this period was allowed for patients with CM [6]. The MEG recording was rescheduled in case of an acute attack during this period or the use of analgesics, triptans, or ergots for any reason in the 48 h before the recording. The temporal relationship between MEG recordings and attacks was determined from the headache diary or through follow-up phone calls.

### MEG recording

Brain activity was recorded using a whole-scalp 306-channel MEG system (Vectorview; Elekta Neuromag, Helsinki, Finland). Four coils representing the head position were placed on the participant’s scalp, positioned in the head coordinate frame with respect to the nasion and two preauricular points by using Cartesian coordinates, and measured with a three-dimensional (3D) digitiser. Approximately 100 additional scalp points were also digitised to obtain an accurate registration. These landmarks enabled further alignment of the MEG and magnetic resonance imaging (MRI) coordinate systems. Individual brain T1 images were acquired using a 3-T MRI system (Discovery 750; GE Medical Systems, WI, USA) with the following parameters: repetition time: 9.4 ms, echo time: 4 ms, recording matrix: 256 × 256 pixels, field of view: 256 mm, and slice thickness: 1 mm.

During the 5-min resting-state MEG recordings with a digitisation rate of 600 Hz, the participants were instructed to close their eyes but remain awake, relax, and perform no explicit task. If a participant fell asleep or had excessive within-run head movement, the recording was stopped and then rerun. Electrooculography (EOG) and electrocardiography (ECG) activities were also acquired simultaneously for offline artefact elimination. A 3-min empty-room recording to capture sensor and environmental noise was applied to calculate the noise covariance for the offline source analysis.

### MEG data preprocessing and analysis

**Fig. 1:**
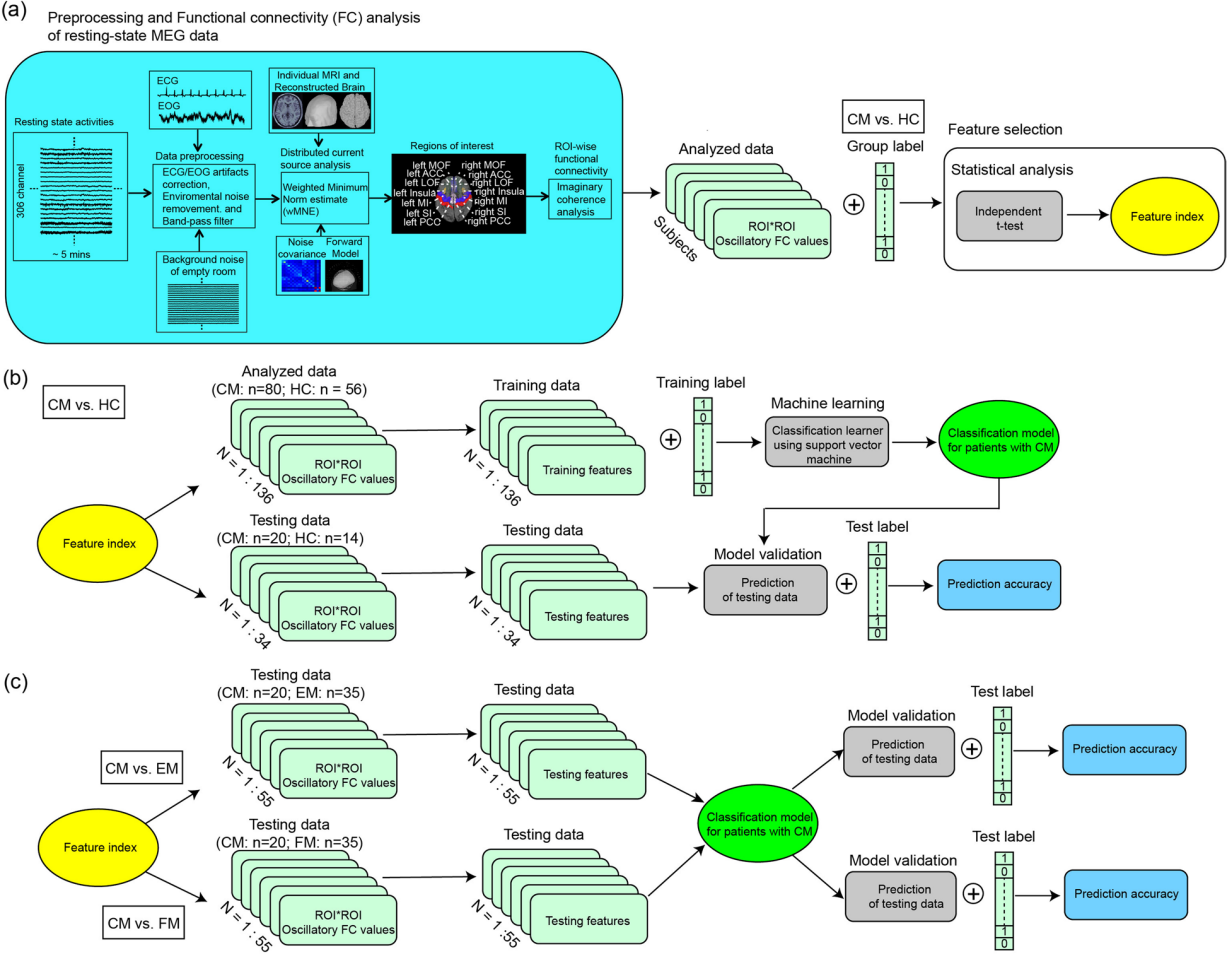
Pipeline of the resting-state MEG and machine learning analysis. CM, chronic migraine; HC, healthy controls; EM, episodic migraine; FM, fibromyalgia

To eliminate the contamination of nonbrain or environmental artefacts from spontaneous resting-state MEG data (Fig. 1), (i) segments containing artefacts from environmental noise were discarded, (ii) notch filters (60 Hz and its harmonics) were used to remove powerline contaminations, and (iii) identified heartbeat and eye blinking events from ECG and EOG data were used to separately define the projectors through principal component analysis [18]. Regarding the T1-weighted brain images, for establishing the cortical model used in source analysis, MR images were automatically reconstructed into a surface model by using BrainVISA (4.5.0, http://brainvisa.info). The anatomical MR images and reconstructed cortical surface were subsequently coregistered with the corresponding MEG data set.

To obtain source-based cortical activation, a distributed source model of the MEG data was established using Brainstorm [19]. The overlapping sphere method [20] and inverse operator were calculated using a depth-weighted minimum norm estimate (MNE) analysis. The source model presents each grid point of the cortical source as a current dipole. The cortical activation dynamics of each participant were finally morphed into a common source space for group analysis. The MNE parameters were described in our previous studies [5, 8, 21]. In the present study, we defined regions of interest (ROIs) in the structural T1 template volume by using Mindboggle cortical parcellation [22], and we selected 26 cortical regions, including the default mode, sensorimotor, visual, salience, and pain networks (details in supplementary Fig. 1).

The oscillatory functional connectivity (FC) between ROIs was calculated from activation dynamics by using the imaginary coherence method at 1–40 Hz with a frequency resolution of 0.586 Hz [6], which essentially measures how the phases between two cortical sources are coupled to each other with minimal crosstalk effects between the sources [23]. The frequency bands of FC were classified as delta (1–4 Hz), theta (5–7 Hz), alpha (8–13 Hz), beta (14–25 Hz), and gamma (26–40 Hz).

### ML analysis

#### Feature selection

Altered oscillatory FC values were associated with the chronification of migraine [6]; thus, utilising these features to establish the predictive migraine model is desirable to obtain high classification accuracy. Feature selection is the process of selecting a subset from the original set of extracted features to increase classification performance with a compact feature subset, which might reduce computational complexity and diminish irrelevant features. Therefore, we applied univariate analyses (independent *t* test) for the factors of the group with the false detection of discovery (FDR) corrections to obtain the most discriminative features, which were also used to construct training and testing data sets (Fig. 1).

#### Classification models and statistical analysis

This study used support vector machine (SVM) algorithms to establish the classification model. SVM algorithms maps input feature vectors into a high-dimensional space to create a linear classification system. By implementing the algorithm with training data, SVM can determine an optimal hyperplane that minimises risks and produces a classification model. The supervised learning approach to train the SVM classifiers decoded two conditions in a pairwise manner (CM vs. HC). The kernel functions and parameters for all classification analyses are listed in Table 1. To avoid overfitting, we trained the models based on a fivefold leave-one-out cross-validation technique. All ML analyses were performed using the ML toolbox from MATLAB software (R2019a).

**Table 1:** Models and parameters of machine classification

SVM	Kernel function	Kernel scale
Linear SVM	Linear	auto
Quadratic SVM	Quadratic	auto
Cubic SVM	Cubic	auto
Fine Gaussian SVM	Gaussian	3
Medium Gaussian SVM	Gaussian	12
Coarse Gaussian SVM	Gaussian	48

*SVM* support vector machine

The performance of each classification model was evaluated based on accuracy, sensitivity, specificity, and the area under the curve (AUC). After the reconstruction and evaluation of the classification models using the training data set, these models were further validated to determine whether identified features are generalisable across various testing data sets, including new testing data sets (CM vs. HC) and other data sets (CM vs. EM; CM vs. FM) (Fig. 1). These data sets were extracted based on the discriminative feature index. The testing data set labels were blinded, and the classification models were applied to the discriminative features without any model training procedure. Predictive accuracy and AUC were obtained for each model. Additionally, to estimate the statistical significance of predictive accuracy, the statistical significance of the observed classification accuracy was estimated using nonparametric permutation tests (10,000 times). The number of permutations in this null distribution that achieved a higher accuracy than the true labels was divided by the total number of permutations. This provided an estimate of the significance of the accuracy relative to chance.

## Results

### Discriminative features of oscillatory connectivity and the classification model for CM

This study included 240 participants—70 HCs, 100 patients with CM, 35 patients with EM, and 35 patients with FM; of them, the data of 56 HCs and 80 patients with CM were included in the training data set, and those of 14 HCs, 20 patients with CMs, 35 patients with EM, and 35 patients with FM were included in the testing data sets. The clinicodemographic characteristics of all participants are summarised in Table 2. In the training data set (HC [*n* = 56] vs. CM [*n* = 80]), the two groups did not differ significantly in terms of age or sex. Anxiety (HADS_A) and depression (HADS_D) scores were higher in the CM group than in the HC group (all *p* < 0.005). In the testing data sets, the groups did not differ significantly in age or sex, except for significantly more women in the FM group than in the CM group (*p* = 0.02). Similar to the training data set, anxiety and depression scores were higher in the CM group than in the HC group (all *p* < 0.005). As expected, patients with CM had more monthly headache days (*p* < 0.001) than those with EM; however, disease duration and severity in the preceding year were comparable between the two groups. MIDAS scores were higher in patients with CM than in those with EM (*p* < 0.05). Notably, the psychometric scores were comparable between the CM, EM, and FM groups.

**Table 2:** Demographics and clinical profiles

	**Training data set**		**Testing data sets**				
	**HC**	**CM**	**HC**	**CM**	**EM**		**FM**
**N**	56	80	14	20	35	**N**	35
**Demographics**						**Demographics**	
Age (years)	41.4 ± 8.3	39.4 ± 11.6	38.3 ± 11.7	36.2 ± 10.5	38.0 ± 11.8	Age (years)	42.5 ± 11.2
Sex	39F/17 M	69F/11 M	11F/3 M	15F/5 M	27F/8 M	Sex	34F/1 M
**Psychometrics**						**Psychometrics**	
HADS_A	4.6 ± 3.5	8.4 ± 4.1	3.7 ± 2.5	9.7 ± 5.4	8.2 ± 4.3	HADS_A	9.8 ± 4.2
HADS_D	3.9 ± 3.0	6.3 ± 3.9	3.7 ± 2.9	7.6 ± 4.3	5.8 ± 3.2	HADS_D	7.6 ± 4.4
**Migraine profile**						**FM profile**	
Headache days (/month)	-	20.1 ± 6.4	-	22.6 ± 6.2	6.6 ± 3.8	WPI	11.0 ± 5.0
Disease duration (months)	-	198.6 ± 144.2	-	182.9 ± 157.5	162.3 ± 129.2	SSS	7.1 ± 2.1
Severity of last year (0–10)	-	6.3 ± 2.1	-	6.1 ± 2.2	5.5 ± 2.1	Painkiller use (days/month)	2.0 ± 3.3
MIDAS scores	-	45.7 ± 61.2	-	51.1 ± 74.9	22.2 ± 28.3	FIQR	39.4 ± 18.1

*HC* Healthy control, *CM* Chronic migraine, *EM* Episodic migraine, *FM* Fibromyalgia, *HADS* Hospital anxiety and depression score, *A* Anxiety, *D* Depression, *MIDAS* Migraine disability assessment, *WPI* Widespread pain index, *SSS* Symptom severity scale, *FIQR* Revised fibromyalgia impact questionnaire

**Fig. 2:**
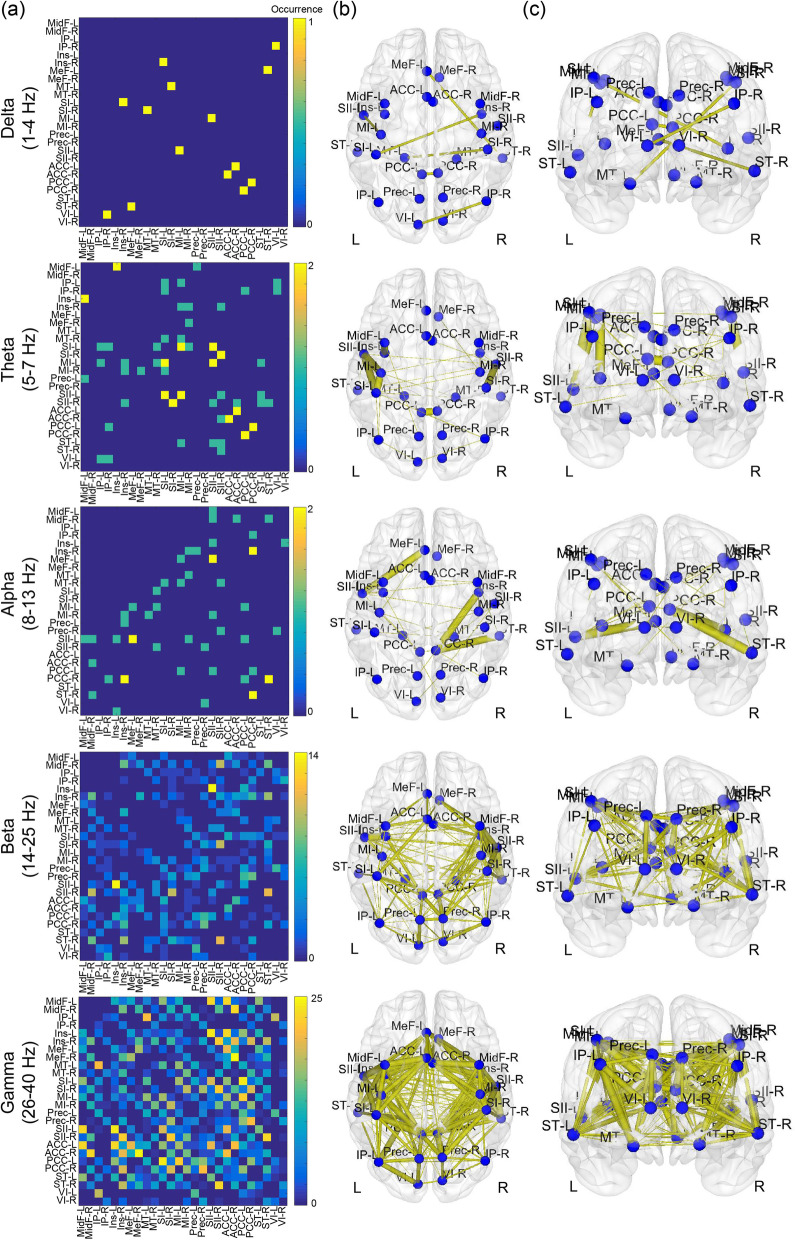
Characteristic features of oscillatory connectivity for differentiating chronic migraine from healthy controls. L, left; R, right

**Fig. 3:**
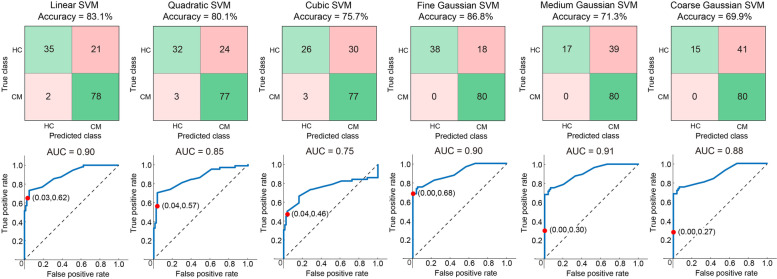
Accuracy and area under the curve (AUC) of machine learning analysis with six kernels for classifying chronic migraine (CM) from healthy controls (HCs) in the training data set. SVM, support vector machine

For feature selection, in the training data set, we obtained 1820 FCs; univariate analysis with FDR corrections revealed that the resting-state oscillatory FCs were significantly different between HC and CM groups. Specifically, patients with CM had decreased oscillatory FCs. These discriminative FCs are illustrated with adjacency matrices from delta to gamma bands (Fig. 2 (a)), and the connections are topographically displayed onto the cortical surfaces with axial and coronal views (Fig. 2 (b) and (c)). The differences were dominantly noted in the beta (19.1% of all significant connections) and gamma (77.4% of all significant connections) bands and primarily located within the following networks: salience (in the insula [7.5%] and anterior cingulate cortex [9.1%]), sensorimotor (in the primary motor cortex [6.6%] and primary [7.3%] and secondary somatosensory cortex [7.1%]), and part of the default mode (in the posterior cingulate cortex [8.1%]) networks. With these discriminative features, classifiers for differentiating CM from HC achieved > 80% accuracy using Linear (accuracy: 83.1%, AUC: 0.9, sensitivity: 0.97, specificity: 0.62), Quadratic (accuracy: 80.1%, AUC: 0.85, sensitivity: 0.96, specificity: 0.57), and Fine Gaussian (accuracy: 86.8%, AUC: 0.9, sensitivity: 1.0, specificity: 0.68) SVMs (Fig. 3).

### Generalizability of the classification model for CM

**Fig. 4:**
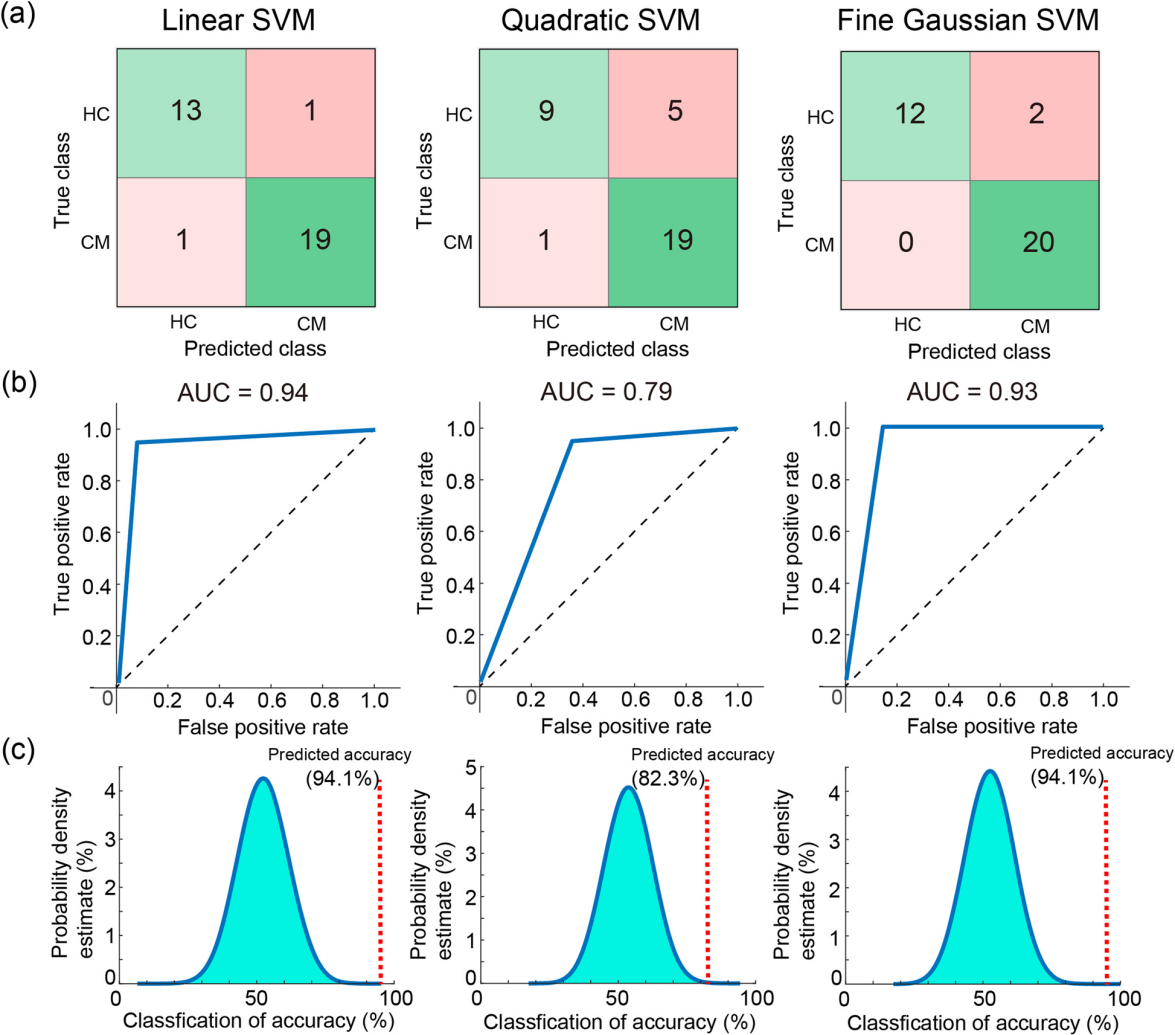
**a** Confusion matrix, (**b**) area under the curve (AUC), and overall prediction accuracy (permutation test) of machine learning analysis with three kernels for classifying chronic migraine (CM) from healthy controls (HC) in the independent testing data set. SVM, support vector machine

**Fig. 5:**
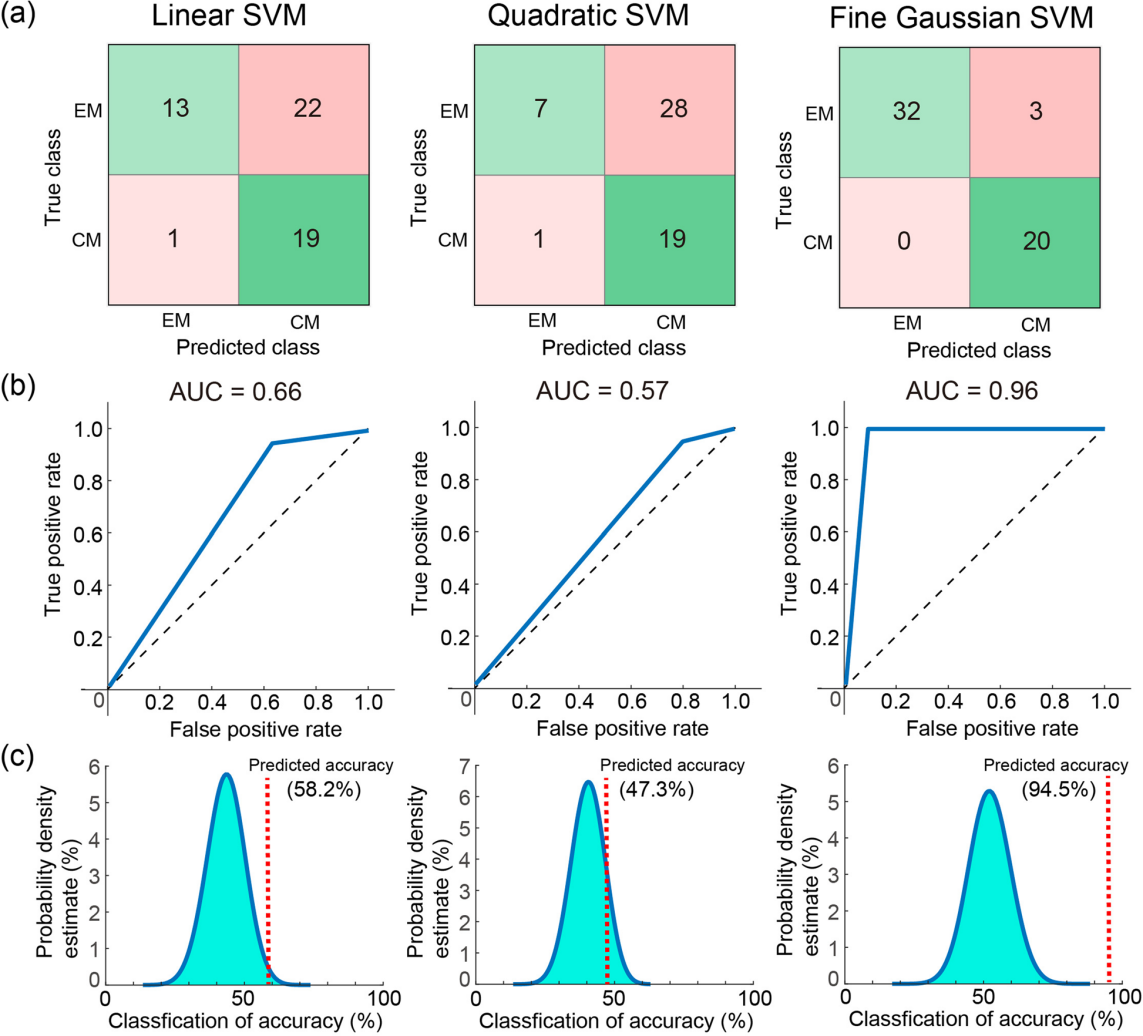
**a** Confusion matrix, (**b**) area under the curve (AUC), and overall prediction accuracy (permutation test) of machine learning analysis with three kernels for classifying chronic migraine (CM) from episodic migraine (EM) in the independent testing data set. SVM, support vector machine

**Fig. 6:**
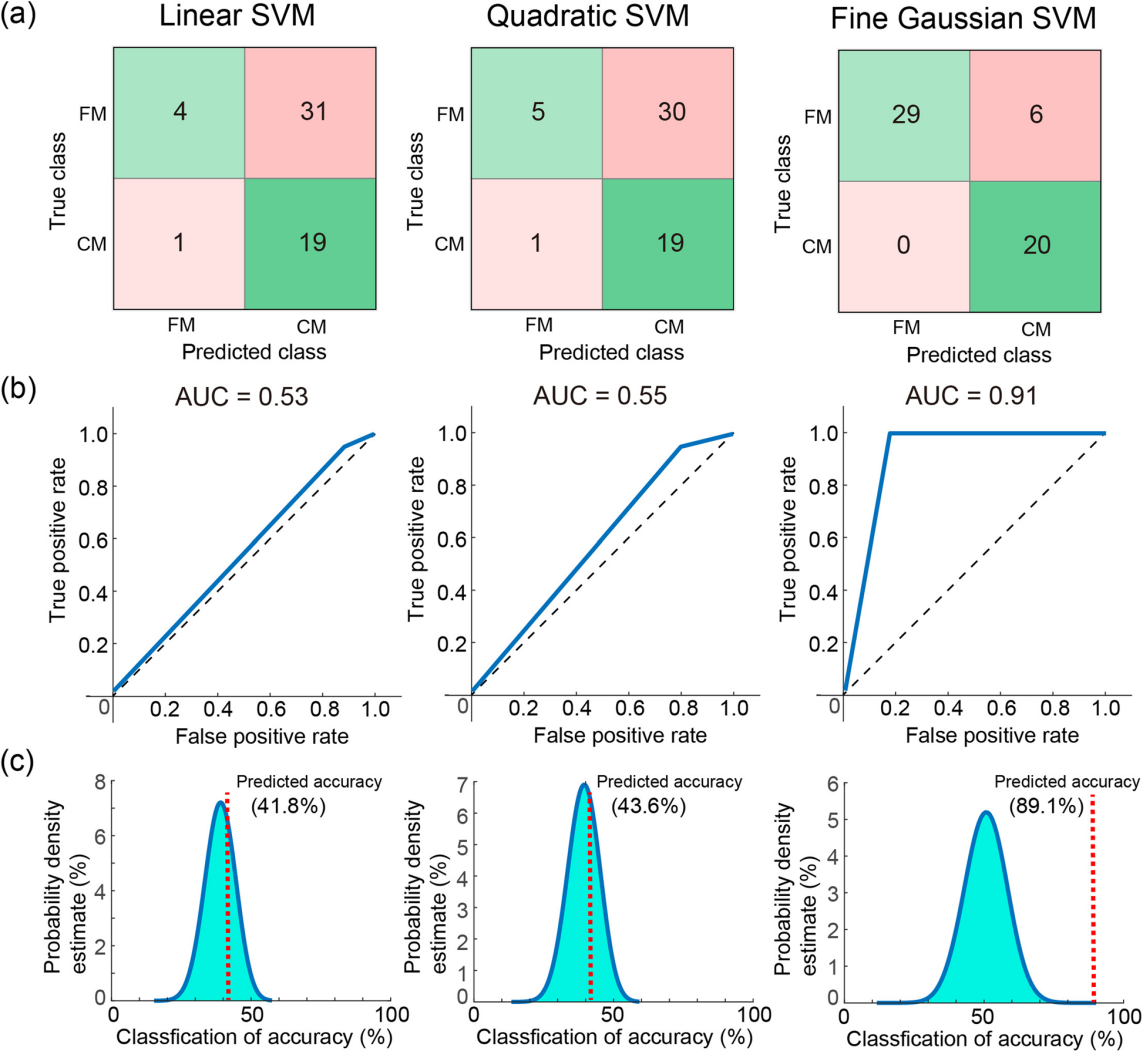
**a** Confusion matrix, (**b**) area under the curve (AUC), and overall prediction accuracy (permutation test) of machine learning analysis with three kernels for classifying the chronic migraine (CM) from fibromyalgia (FM) in the independent testing data set. SVM, support vector machine

We further examined the generalizability of the classification model with the three SVMs by using an independent data set of 20 patients with CM and 14 HCs. The model exhibited good performance using Linear (accuracy: 94.1%, AUC: 0.94, sensitivity: 0.95, specificity: 0.93, *p* < 0.0001), Quadratic (accuracy: 82.3%, AUC: 0.79, sensitivity: 0.95, specificity: 0.64, *p* = 0.0004), and Fine Gaussian (accuracy: 94.1%, AUC: 0.93, sensitivity: 1.0, specificity: 0.86, *p* < 0.0001) SVMs (Fig. 4). The model performance in classifying different headache subtypes was assessed using a data set of 20 patients with CM and 35 patients with EM. The Fine Gaussian SVM achieved high performance (accuracy: 94.5%, AUC: 0.96, sensitivity: 1.0, specificity: 0.91, *p* < 0.0001) (Fig. 5). Finally, the model performance in identifying CM compared with other chronic pain disorders was assessed using a data set of 20 patients with CM and 35 patients with FM. The Fine Gaussian SVM (Fig. 6) achieved favourable results (accuracy: 89.1%, AUC: 0.91, sensitivity: 1.0, specificity: 0.83, *p* < 0.0001). In summary, these findings indicated that our model had good generalizability for identifying patients with CM in an independent data set.

## Discussion

In this study, by utilising resting-state MEG recording data to develop SVM models, we discovered the FCs of spontaneous neuromagnetic activities that could identify patients with CM. The discriminative features were principally deciphered from the functional interactions among salience, sensorimotor, and part of the default mode networks. The classification model with these features exhibited excellent performance in differentiating patients with CM from HC (training data set: accuracy = 86.8%, AUC = 0.9; testing data set: accuracy = 94.1%, AUC = 0.93). Moreover, this model also performed well in distinguishing patients with CM from those with EM (accuracy: 94.5%, AUC: 0.96) as well as from those with FM (accuracy: 89.1%, AUC: 0.91). These findings imply that resting-state MEG signatures can be specific and reliable for identifying patients with CM.

### Characteristic features of oscillatory connectivity in CM

Altered oscillatory connectivity was dominantly noted in the beta and gamma bands among salience, sensorimotor, and default mode networks; this indicated that patients with CM had altered oscillatory synchrony, impairing flexible routing of information across brain areas [24, 25]. Consistent with previous MEG [6] and EEG findings [26, 27], our data revealed decreased FC at distinct frequency bands in patients with migraine. This corresponds to the notion that pain is associated with complex temporal–spectral–spatial synchrony patterns of brain activity [11], which are abnormal in patients with chronic pain [6, 28–30]. Although the findings implied no substantial relationship of the specific brain oscillation with pain processing [31], pain occurring with the integration of nociceptive and contextual information is mediated by feedforward and feedback processes in the brain [32], which are, respectively, engaged in gamma and alpha/beta oscillations [11]. Correspondingly, aberrant cortical processing for CM was mainly identified with beta and gamma oscillations in this study. Beta activities in animal models of chronic pain changed over the primary somatosensory and frontal cortex [30, 33, 34]. Gamma oscillation before stimulus onset was correlated with pain perception [35], and abnormal gamma oscillation has been associated with neurological and psychiatric symptoms, including ongoing pain, resulting from thalamocortical dysrhythmia [36, 37]. Taken together, the oscillatory connectivity selected from this study by using a statistical approach adequately represents the cortical-level neuropathological characteristics in patients with CM.

The main hubs in the cortical areas exhibiting the alterations for CM included the insula, anterior cingulate cortex (ACC), primary motor, primary somatosensory, secondary somatosensory, and posterior cingulate cortex areas, which are located in the salience, sensorimotor, and default mode networks. In accordance with previous neurophysiological findings [6, 26, 27], decreased FC within the frontal–central, central–parietal, temporal-parietal, or pain-related networks was noted in patients with migraine. Intriguingly, these cortical areas have shown their crucial roles in sensory-discriminative and affective-motivational pain, associated with headache severity. Activities in the sensorimotor cortex were associated with pain intensity [38]. FC in ACC was related to migraine chronification [6]. The subjective perception of pain intensity was related to bilateral insula activation [39, 40]. Moreover, the posterior cingulate cortex is engaged in both pain sensitivity and integration of inputs from different sensory modalities (multisensory integration), which is declined in patients with migraine [41]. Theta connectivity between the insula and default mode network exhibited associations with FM severity [42]. Altered activities in the salience, sensorimotor, and default mode networks have also been noted in patients with chronic pain [28, 29, 43]. Notably, gamma oscillation was elicited to encode afferent sensory information over the sensory cortex; however, after long-lasting pain, it appeared over brain areas encoding emotional–motivational processing and dominated the processing and perception of pain [11, 44]. This implies that the pain process not only is spatially distributed over the brain network but also dynamically recruits brain areas for the adaptive integration of nociceptive, cognitive, and affective processing, which are apparently aberrant in patients with chronic pain. Therefore, the characteristic features of FC among the cortical networks that were observed in this study might serve as pivotal signatures to identify CM.

In this resting-state MEG study, the participants were instructed to close their eyes but remain awake, relax, and perform no explicit task. Thus, the recorded MEG signals were spontaneous cortical activities and originated from the intrinsic brain networks, which were distinct from the evoked activities from pain-related emotional stimuli [45]. Besides, Hepschke and colleagues suggested that rhythmical brain activity in the primary visual cortex is both hyperexcitable and disorganized in visual snow syndrome [46]. Notably, in our study, no migraine patient was diagnosed with visual snow syndrome; nevertheless, in our previous and present studies, patients with CM was characterized with hyperexcitability or disinhibition in visual and somatosensory cortices [5, 8, 47–49] and aberrant functional connectivity in pain-related areas [6]. Common altered pathophysiological mechanisms might exist between patients with and without visual snow syndrome, but the dominant involved cortical areas should differ from both.

### Identifying patients with CM using ML

The classification model of the present study exhibited favourable performance in differentiating patients with CM from HC, EM, and FM (all accuracy > 86% and AUC > 0.9), indicating the generalizability of this model on the prediction of patients with CM from the populations of migraine (CM vs. EM) and from chronic pain disorders (CM vs. FM). By using advanced neuroimaging approaches, studies have reported that the brain networks during the stimulus-evoked or resting-state conditions were altered in patients with migraine; however, whether these functional or structural features can help achieve migraine classification using ML algorithms has been somewhat unclear. The CM classification model developed by Schwedt and colleagues [50] by using brain structural imaging data achieved accuracies of 86.3% (CM [*n* = 15] vs. HC [*n* = 54]) and 84.2% (CM [*n* = 15] vs. EM [*n* = 51]). Resting-state FC data obtained from functional MRI achieved 86.1% accuracy in discriminating the brain of patients with migraine [*n* = 58] from that of HC [*n* = 50] [51]. The combination of functional and structural MRI data achieved 83.67% accuracy in discriminating the patients with migraine (*n* = 21) from HC (*n* = 28) [52]. These three studies also exhibited the prominent role of the pain-related areas, especially the insula and cingulate cortex, in the identification of patients with migraine. However, the parameters of somatosensory-evoked high-frequency oscillations achieved somewhat higher accuracies of 89.7% (HC [*n* = 15] vs. ictal migraine [*n* = 13]) and 88.7% (HC [*n* = 15] vs. interictal migraine [*n* = 29]) in identifying patients with migraine [53]. Nevertheless, these studies have limited reliability and generalizability because of small sample sizes, lack of independent data to validate the findings, and lack of evaluation of whether these discriminative features specifically characterised the migraine. Our study overcame these shortcomings by recruiting 240 participants and dividing them into subgroups of training or independent testing data sets; moreover, the factors of different migraine spectrum and other chronic pain disorders were considered.

One promising study by Tu and colleagues [54] identified, cross-validated, independently validated, and cross-sectionally validated the fMRI-based neural marker and claimed that the functional connections within the visual, default mode, sensorimotor, and frontoparietal networks had 84.2%–91.4% accuracy in identifying patients with EM from HC and 73.1% accuracy in identifying patients with EM from those with other chronic pain disorders (FM and chronic low back pain). In addition to the fundamental difference between fMRI and MEG techniques, oscillatory connectivity analysis in this study (MEG with frequency bands of 1–40 Hz neural activities compared with fMRI confined within < 0.01 Hz blood oxygen level–dependent activities) might contribute to improved accuracies of migraine classification. Rocca et al. [55] claimed that classification performance using fMRI data might be influenced by the factors of scanners and acquisition protocols. MEG-based electrophysiological features obtained from direct neural recordings with fine spatial resolution and excellent temporal resolution, which assess the oscillatory connectivity between relevant cortical areas, might eventually help diagnose patients with CM. Moreover, the frequency- and region-specific MEG signatures used in this model might represent the targeting candidates of migraine neuromodulation using transcranial magnetic and direct current stimulation.

### Limitations

Our study had several limitations. First, all participants were naïve to any migraine preventive medication, precluding the determination of whether our model can be generalised to patients taking such treatments. Second, the presence of background or interval headache, but not ictal attack, was allowed for patients with CM. Although our study noted the dynamic brain excitability within the migraine cycle in patients with EM [56], the longitudinal day-to-day dynamics of FC between brain networks in patients with CM remain unresolved, which leads to an unsettled question of how the classification model performs for the factor of headache status. Third, we demonstrated the reliability and generalizability of using MEG-based electrophysiological features. However, whether the aforementioned brain signatures are shared with primary headaches or chronic pain disorders other than CM, EM, and FM remains unknown. Finally, MEG is expensive and not easily accessible, thus limiting the clinical application of this classification model; therefore, translations between MEG and high-density EEG are warranted.

## Conclusion

These resting-state MEG-based electrophysiological features yield oscillatory connectivity to differentiate patients with CM from those with a different type of migraine and chronic pain disorders with good reliability and generalizability. This classification model may help in an objective and individualised diagnosis of migraine. However, the present findings need further studies to examine their diagnostic value in real-world clinical settings and to justify their link to the CM pathophysiology.

## Supplementary Information

**Additional file 1: Supplementary Fig. 1.** Regions of interest used in the study. Abbr., abbreviation; P, posterior; A: anterior; L, left; R, right.

## Abbreviations

EM: Episodic migraine
CM: Chronic migraine
ML: Machine learning
MEG: Magnetoencephalography
HC: Healthy controls
FM: Fibromyalgia
HADS: Hospital Anxiety and Depression Scale
MIDAS: Migraine Disability Assessment Scale
3D: Three-dimensional
MRI: Magnetic resonance imaging
EOG: Electrooculography
ECG: Electrocardiography
MNE: Minimum norm estimate
ROIs: Regions of interest
FC: Functional connectivity
FDR: False detection of discovery
SVM: Support vector machine
AUC: Area under the curve
ACC: Anterior cingulate cortex
