# Supplementary figures and images for: Resting-state magnetoencephalographic oscillatory connectivity to identify patients with chronic migraine using machine learning
Source: J Headache Pain. 2022 Oct 3;23(1):130. doi: 10.1186/s10194-022-01500-1 (PMC9531441; doi:10.1186/s10194-022-01500-1)

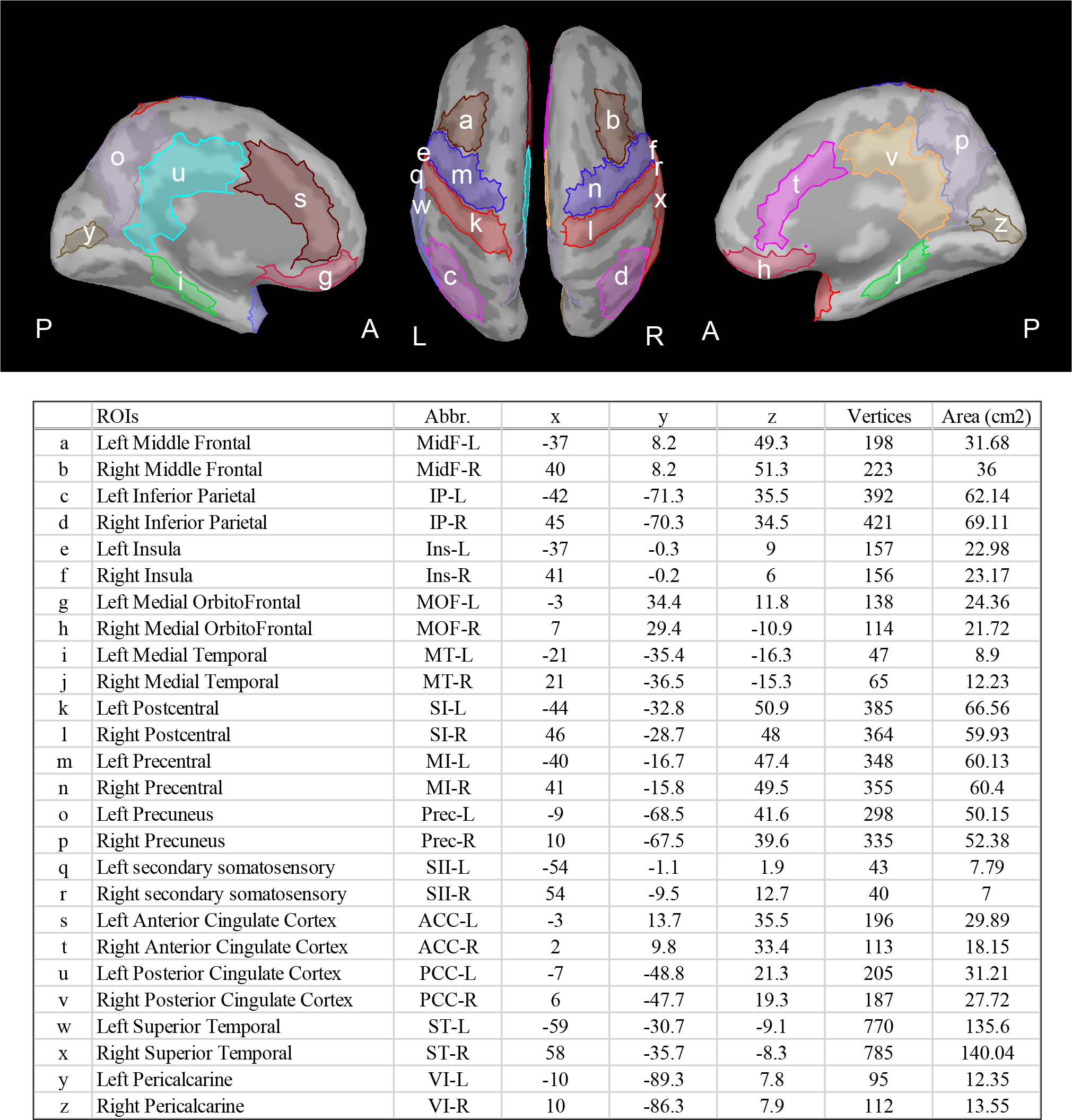

Supplement: Supplementary file 1 — Additional file 1: Supplementary Fig. 1. Regions of interest used in the study. Abbr., abbreviation; P, posterior; A: anterior; L, left; R, right. [file 10194_2022_1500_MOESM1_ESM.jpg]
